# Characterization of eight novel proteins with male germ cell-specific expression in mouse

**DOI:** 10.1186/1477-7827-6-32

**Published:** 2008-07-24

**Authors:** Namhoe Baek, Jong-Min Woo, Cecil Han, Eunyoung Choi, Inju Park, Do Han Kim, Edward M Eddy, Chunghee Cho

**Affiliations:** 1 Department of Life Science and Research Center for Biomolecular Nanotechnology, Gwangju Institute of Science and Technology, Gwangju 500-712, Korea; 2 Gamete Biology Section, Laboratory of Reproductive and Developmental Toxicology, National Institute of Environmental Health Sciences, National Institutes of Health, Research Triangle Park, North Carolina 27709, USA

## Abstract

**Background:**

Spermatogenesis and fertilization are highly unique processes. Discovery and characterization of germ cell-specific genes are important for the understanding of these reproductive processes. We investigated eight proteins encoded by novel spermatogenic cell-specific genes previously identified from the mouse round spermatid UniGene library.

**Methods:**

Polyclonal antibodies were generated against the novel proteins and western blot analysis was performed with various protein samples. Germ cell specificity was investigated using testes from germ cell-less mutant mice. Developmental expression pattern was examined in testicular germ cells, testicular sperm and mature sperm. Subcellular localization was assessed by cell surface biotin labeling and trypsinization. Protein localization and properties in sperm were investigated by separation of head and tail fractions, and extractabilities by a non-ionic detergent and urea.

**Results:**

The authenticity of the eight novel proteins and their specificity to spermatogenic cells were confirmed. In examining the developmental expression patterns, we found the presence of four proteins only in testicular germ cells, a single protein in testicular germ cells and testicular sperm, and three proteins in the testicular stages and mature sperm from the epididymis. Further analysis of the three proteins present in sperm disclosed that one is located at the surface of the acrosomal region and the other two are associated with cytoskeletal structures in the sperm flagellum. We name the genes for these sperm proteins Shsp1 (Sperm head surface protein 1), Sfap1 (Sperm flagellum associated protein 1) and Sfap2 (Sperm flagellum associated protein 2).

**Conclusion:**

We analyzed eight novel germ cell-specific proteins, providing new and inclusive information about their developmental and cellular characteristics. Our findings will facilitate future investigation into the biological roles of these novel proteins in spermatogenesis and sperm functions.

## Background

Male germ cell development involves successive mitotic (spermatogonia), meiotic (spermatocyte) and postmeiotic phases (spermatids). Spermatogonial stem cells, located around the outer region next to the basal lamina surrounding the seminiferous tubules in the testis, divide mitotically to produce primary spermatocytes. These cells continue through the first meiotic division to become haploid secondary spermatocytes. During this division, random assortment of paternal or maternal chromosomes and chromosomal crossover take place, generating the genetic diversity of the gametes. Secondary spermatocytes rapidly enter the second meiotic division to produce spermatids. These haploid spermatids are then remodeled into sperm by spermiogenesis. During this period, spermatids begin to grow tails and their chromatin undergoes packaging, inactivating transcription from the haploid male genome. The acrosome derived from the Golgi apparatus envelopes the anterior portion of the condensed nucleus.

Since the development of sperm specialized for fertilization is a unique process that occurs only in testis, gaining an understanding of spermatogenesis and fertilization requires identification and characterization of genes specifically expressed in testicular germ cells. Previously, we analyzed the mouse spermatocyte and round spermatid UniGene libraries containing 2124 and 2155 gene-oriented transcript clusters, respectively [1,2]. UniGene is a NCBI database containing an extensive collection of information about sets of transcript sequences. In particular, the UniGene database is a useful resource for identifying tissue- and cell type-specific gene transcripts. These studies revealed that the proportions of testis-specific genes in the spermatocyte and round spermatid UniGene libraries are 11% (230 genes) and 22% (467 genes), respectively. Notably, more than half of the testis-specific genes were found to be unknown. The unexplored testis-specific genes were analyzed further. Through systematic *in silico *and *in vitro *analyses these genes were narrowed down to 24 (the spermatocyte UniGene study) and 28 (the round spermatid UniGene study) genuine genes abundantly and specifically transcribed in mouse testis. Based on *in silico *information, a number of these genes were predicted to be involved in diverse functions such as transcriptional regulation, nuclear integrity, cell structure and metabolism. Further, some of the genes identified from the round spermatid UniGene library were investigated at the protein level. Remarkably, one of these novel proteins turned out to be a sperm acrosomal protein with a trypsin-like serine protease domain [2].

Here, as an ongoing study on the novel spermatogenic cell-specific genes, we investigated eight proteins encoded by the novel genes discovered previously from the mouse round spermatid UniGene library [1]. The authenticity and germ cell specificity of these genes were confirmed at the protein level. We obtained original findings on the developmental expression pattern and localization of these eight novel proteins. In particular, three novel proteins were found to be present in mature sperm. Our data revealed that one of the other proteins is located at the surface of the acrosomal region and two are associated with cytoskeletal structures in the sperm tail. This study presents the first characterization of these eight novel spermatogenic cell-specific genes, with potential roles in spermatogenesis and fertilization, at the protein and cellular levels.

## Methods

### Antibody production

**Figure 1:**
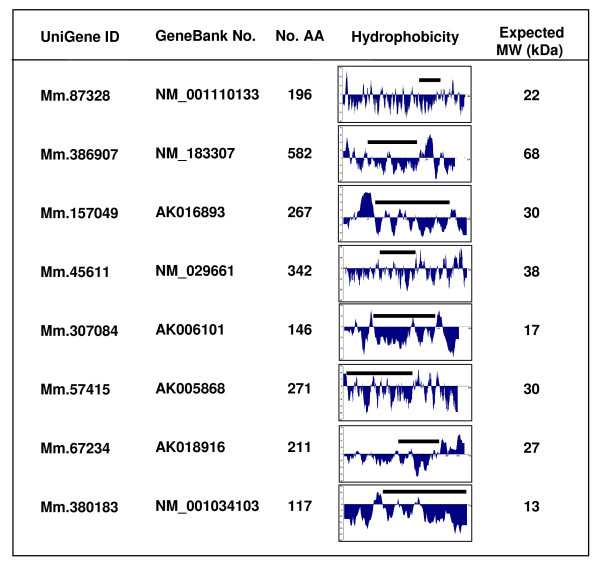
***In silico *****information on the eight novel genes.** GeneBank accession numbers for the cDNA sequences of the novel genes are listed. Amino acid sequences, hydrophobicity and expected molecular weights are based on coding regions predicted by selecting the longest open reading frames deduced from cDNA sequences. The bars indicate regions corresponding to antigens used for antibody generation. AA, amino acids; MW, molecular weight.

To produce glutathione S-transferase (GST) fusion proteins, PCR products corresponding to the hydrophilic regions of the eight novel proteins (amino acids 61–171 for Mm.87328, 379–460 for Mm.386907, 61–180 for Mm.157049, 101–200 for Mm.45611, 40–119 for Mm.307084, 10–180 for Mm.57415, 97–165 for Mm.67234 and 41–117 for Mm.380183) were generated using gene-specific primers (Fig. 1). After restriction digestion, the PCR products were ligated with a pGEX-5X-2 vector (Amersham Pharmacia). The resulting constructs were expressed in *E. coli *BL21. GST fusion proteins subsequently were isolated with glutathione Sepharose 4B and used as antigens for production of rabbit polyclonal antibodies. All the antibodies were affinity purified using their corresponding proteins and the AminoLink Immobilization kit (Pierce). Monoclonal anti-mouse a disintegrin and metalloprotease 2 (ADAM2) was purchased from Chemicon.

### Western blot analysis

Proteins denatured by boiling for 5 min in the presence of 3% SDS and 5% β-mercaptoethanol were separated by SDS-PAGE, and then transferred onto a polyvinylidene difluoride (PVDF) membrane (0.2 μm; Bio-Rad Laboratories). After blocking with 5% nonfat dry milk in TBS-T (TBS: 50 mM Tris-HCl, pH 7.5, 150 mM NaCl and 0.1% Tween-20), the blots were incubated with primary antibodies for 1 h 30 min at room temperature, followed by three washes for 10 min with TBS-T, and then incubated with alkaline phosphatase-conjugated secondary antibodies (Jackson Immunoresearch) for 1 h at room temperature. Alkaline phosphatase activity was detected by NBT/BCIP (Promega Biotech).

### Preparation of testicular cells, testicular sperm, and mature sperm

Testicular (spermatogenic) cells and testicular sperm from adult mice (3–6 months old) were prepared as described [3]. Briefly, the cells are isolated by suspension in 52% isotonic Percoll (Pharmacia) and centrifugation for 10 min (27,000 g, 10°C), and resuspended in Mg^2+^-Hepes buffer. Testicular cells are spermatogenic cells corresponding to spermatogonia, spermatocytes and round spermatids. The population of testicular sperm includes a small fraction of elongating and condensing spermatids and a larger number of fully developed sperm. Post-testicular, mature sperm from the cauda epididymis and vas deferens were directly released into PBS. To prepare acrosome reacted sperm, sperm from the cauda epididymis and vas deferens were induced to undergo acrosome reaction by calcium ionophore A23187 (Sigma) at a concentration of 5 μg/ml at 37°C for 1 h under 5% CO_2 _in air. The sperm suspension was centrifuged twice at 800 g for 10 min to remove contaminants. The collected sperm were resuspended directly in 1× SDS sample buffer (3% SDS), followed by boiling for 5 min, or lysed with a non-ionic detergent (1.0% Triton X-100 in PBS) for 40 min on ice in the presence of 1× protease inhibitor cocktails (Calbiochem). The lysates were centrifuged for 10 min at 12000 g. The supernatants from the lysates were mixed with 2× SDS sample buffer (6% SDS), were reduced with 5% β-mercaptoethanol, boiled for 5 min and subjected to western blot analysis.

### Cell surface biotinylation and trypsinization

Sperm from the cauda epididymis and vas deferens were kept at room temperature for 30 min in PBS containing 1 mg/ml sulfo-NHS-LC-biotin (Pierce) or kept on ice for 30 min in cold PBS containing 0.5 mg/ml trypsin (Sigma). The biotinylated sperm were washed three times with PBS and the trypsinized sperm were washed three times with PBS containing 1× protease inhibitor cocktail (CALBIOCHEM). The biotinylated and trypsinized sperm were mixed with 2× SDS sample buffer (6% SDS), reduced with 5% β-mercaptoethanol, boiled for 5 min and subjected to western blot analysis.

### Sperm fractionation

Sperm from the cauda epididymis and vas deferens were directly released into PBS and washed with PBS. Collected sperm were resuspended in 2.5 ml of PBS containing 2 mM EGTA and 1 mM β-mercaptoethanol. After sonication for four 10 s bursts, an equal volume of 1.8 M sucrose was added and the suspension was layered over a discontinuous sucrose gradient containing equal volumes of 2.05 M and 2.2 M sucrose solutions. The sample was centrifuged at 100,000 g at 4°C for 16 h. The sperm heads and tails were then collected from the pellets at the bottom and the middle of the 2.05 M sucrose layer, respectively. The heads and tails were washed with PBS and the samples were subjected to western blot analysis.

### Extraction analysis of sperm proteins

Sperm from the cauda epididymis and vas deference were directly released into PBS, and washed three times with PBS. The sperm were lysed in a lysis buffer (10 mM Tris-Cl, pH 8.0, 150 mM KCl, 5 mM MgCl_2_, 0.5 mM EDTA) containing either 2, 3, 4 or 6 M urea, or 1% Triton X-100 on ice for 2 h, except for the lysis buffer containing 6 M urea, which was incubated at room temperature. After incubation, the soluble and insoluble fractions were separated by centrifugation at 10,000 g for 15 min, and each fraction was subjected to western blot analysis.

## Results

### Generation of antibodies and expression pattern of novel proteins

Of 28 novel testis-specific genes investigated at the transcript level in our previous study [1], eight genes were analyzed at the protein level in this study (Fig. 1). They are Mm.87328, Mm.386907, Mm.157049, Mm.45611, Mm.307084, Mm.57415, Mm.67234 and Mm.380183 (Fig. 1). The UniGene ID numbers for three of the genes have been changed. The original ID numbers for Mm.386907, Mm.57415 and Mm.380183 are Mm.84974, Mm.273313 and Mm.46105, respectively, in our previous study [1]. It should be noted that expressed sequence tags (ESTs) for some (Mm.87328, Mm.386907, Mm.157049 and Mm.57415) of the eight genes are found in non-testicular tissues in the current UniGene database. In the cases of Mm.87328, Mm.386907 and Mm.157049, however, the proportion of the number of testicular ESTs to that of non-testicular ESTs is exceedingly high (>75%). For Mm.57415, currently at least two different transcripts are listed erroneously in the database. The GenBank accession number of a genuine transcript/gene investigated in the previous and present studies for Mm.57415 is AK005868 which has ESTs only in testis. Most importantly, transcription of all the four genes was analyzed *in vitro *and found to be specific to testis in the previous study [1]. According to our further *in silico *analysis and information in the database, none of the proteins encoded by the eight genes is predicted to contain specific motifs/domains and belong to a known protein family. Thus, they are not assigned with potential functions.

**Figure 2:**
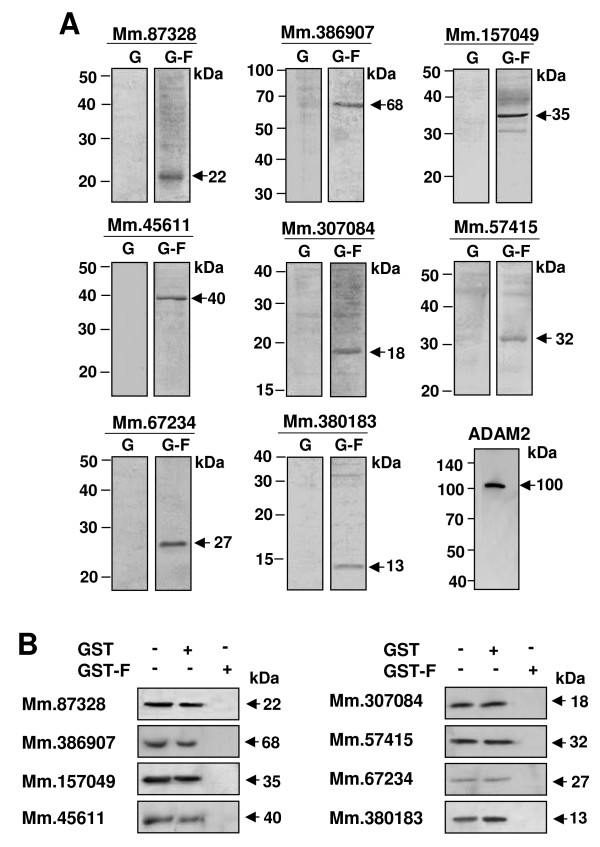
**Specificity of antibodies to the novel proteins.** (A) Testicular germ cells were isolated from testis, boiled in 3% SDS with 5% β-mercaptoethanol, subjected to SDS-PAGE and blotted with antibodies. Two antibodies were used for the test of antibody specificity to each protein. The first one is a control antibody affinity-purified against GST (G) protein from each serum. The second one is a test antibody affinity-purified against each GST fusion protein (G-F) used for immunization and serum production. ADAM2, known to exhibit spermatogenic cell-specific expression, was included as a control. (B) GST or GST fusion (GST-F) proteins were added to a buffer containing the first antibodies during the incubation of immune blot analysis.

**Figure 3:**
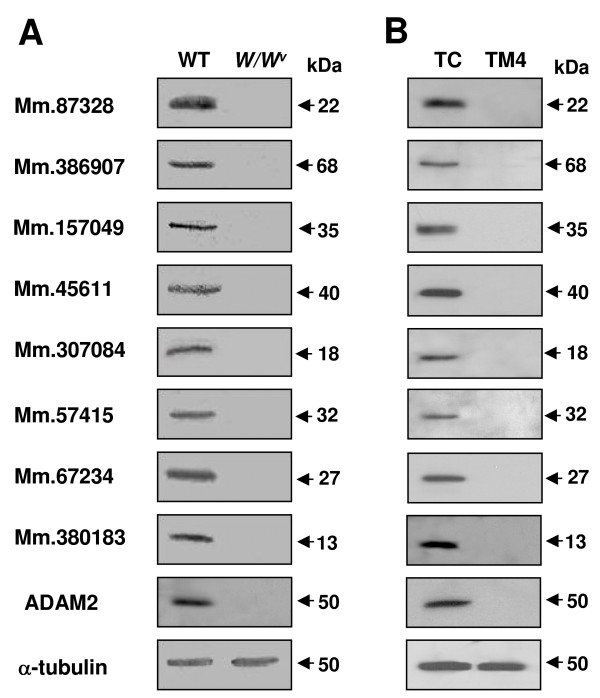
**Germ cell-specific expression of the novel proteins.** Testes from *W/W*^*v *^mutant mice (A) and a Sertoli cell line, TM4 (B), were subjected to protein extraction and western blot analysis using the specific antibodies. Total proteins from the testes were extracted by boiling the minced tissues in a protein sample buffer containing 3% SDS and 5% β-mercaptoethanol. ADAM2, with spermatogenic cell-specific expression, was included as a control. WT, wild type; TC, testicular (spermatogenic) cells.

We generated antibodies against proteins encoded by eight novel genes. GST fusion proteins containing the various regions of proteins deduced from the cDNA sequences of the novel genes were produced and used to generate polyclonal sera. Antibodies were affinity-purified against GST alone (as a control) and the cognate GST fusion protein. To test the specificity of the antibodies to the novel proteins and determine whether the novel proteins are present in testis, we carried out western blot analysis using the two antibodies for each novel protein on testicular spermatogenic cells. As shown in Figure 2A, the antibodies to the GST fusion proteins, but not the antibodies to GST alone, recognized distinct bands of sizes comparable to the predicted molecular weights (Fig. 1). Moreover, when the GST fusion proteins used for generating the antibodies were added during incubation with the first antibodies in the immnunoblot, they blocked binding of the antibodies to the bands in all of the eight proteins (Fig. 2B). These data suggest the specificity of the antibodies and authenticity of the novel proteins. To further determine whether the novel proteins are spermatogenic cell-specific in the testis, western blot analysis was performed with the protein extracts of the germ cell-lacking testes of the *W/W*^*v *^mutant mice [4] and a Sertoli cell line derived from the testicular somatic cells. We found that none of the proteins were expressed in the mutant mice (Fig. 3A) and Sertoli cells (Fig. 3B), strongly suggesting that all of the eight novel proteins are expressed only in germ cells in testis.

**Figure 4:**
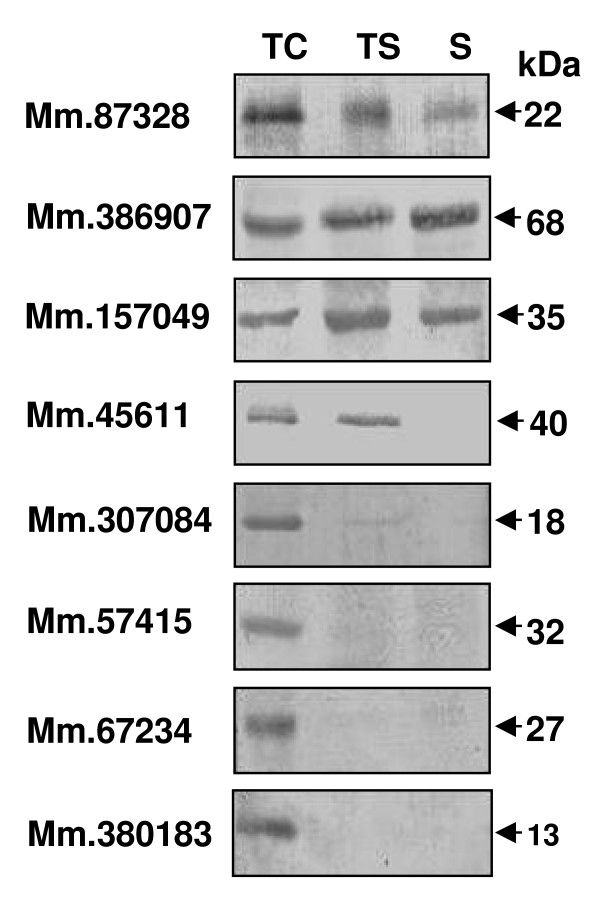
**Developmental expression pattern of the novel proteins.** Cells at different developmental stages were isolated, boiled in 3% SDS with 5% β-mercaptoethanol, subjected to SDS-PAGE and blotted with the specific antibodies. TC, testicular (spermatogenic) cells; TS, testicular sperm; S, mature sperm from the epididymis and vas deferens.

To investigate the developmental expression patterns of the eight novel proteins during spermatogenesis, western blot analysis was performed with testicular cells, testicular sperm, and mature sperm from the cauda epididymis and vas deferens. We found that four of the proteins (Mm.307084, Mm.57415, Mm.67234 and Mm.380183) are present only in testicular cells; the Mm.45611 protein exists in testicular cells and sperm but not mature sperm; and the other three proteins (Mm.87328, Mm.386907 and Mm.157049) are present in all of the cell types. None of these proteins had changes in their molecular masses during spermatogenesis (Fig. 4). Thus, these results suggest differential regulation of the eight proteins during male germ cell development. It should be noted that, to confirm and further examine the developmental expression and localization of the proteins, we performed immunohistochemical analysis in paraffin section of mouse testis and immunofluorescence analysis with isolated germ cells from the testis and epididymis. Unlike immunoblot analysis, none of the antibodies displayed inmmunoreactivity in these experiments.

### Localization of novel proteins present in mature sperm

**Figure 5:**
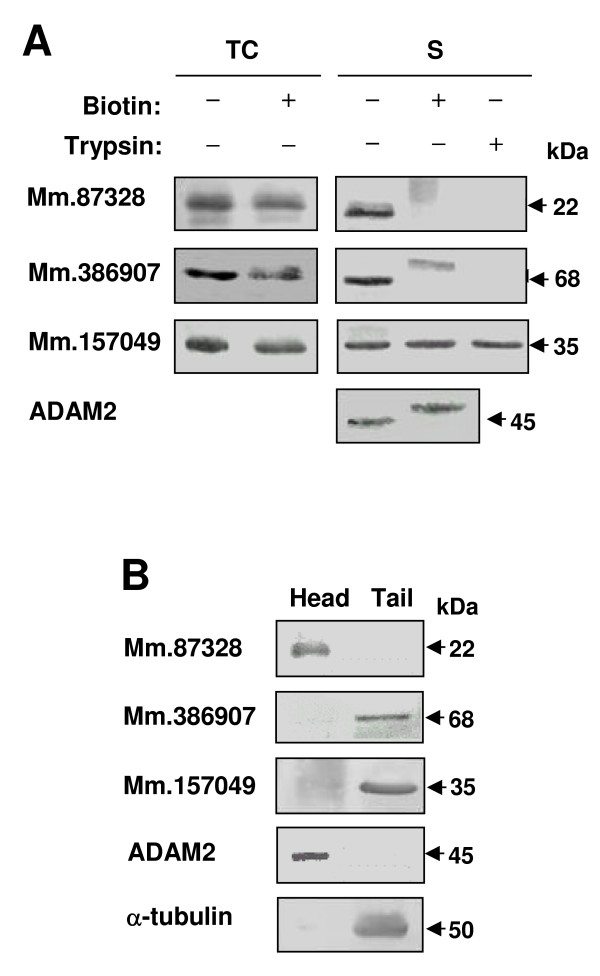
**Localization of the Mm.87328, Mm.386907 and Mm.157049 proteins.** (A) Testicular cells, and sperm from cauda epididymis and vas deferens were (+) or were not (-) treated with biotin or trypsin and then subjected to western blot analysis. ADAM2, known to be located at the sperm surface, was included as a control. TC, testicular (spermatogenic) cells; S, mature sperm from the epididymis and vas deferens. ADAM, a disintegrin and metalloprotease. (B) Sperm from the epididymis and vas deferens were fractionated into heads and tails. These fractions were subjected to western blot analysis. ADAM2 localized on sperm head and α-tubulin composing the axoneme in the sperm flagellum were used as control proteins.

Because three of the novel proteins (Mm.87328, Mm.386907 and Mm.157049) were found to exist in mature sperm, it is possible that they are related to sperm function and fertilization. As an initial step to investigate the proteins in these reproductive processes, we examined subcellular localization of the proteins. As shown in Figure 5A, cell surface labeling and trypsinization experiments were conducted. A disintegrin and metalloprotease 2 (ADAM2), a sperm surface protein, was included as a reference protein [5]. Surface labeling of testicular cells with biotin did not cause any change of the three novel proteins. However, sperm surface labeling with biotin resulted in alteration of two proteins. The Mm.87328 protein was found to disappear or be present in a blurred band after biotinylation in the western blot result. In addition, the molecular weight of the Mm.386907 protein was increased by biotinylation. A probable reason for disappearance of the Mm.87328 protein is that an epitope for antibody binding in the protein is masked by biotinylation. Consistent with these results, both the Mm.386907 and Mm.87328 proteins, but not the Mm.157049 protein, were affected by sperm surface trypsinization (Fig. 5A). Thus, these results suggest the localization of the two novel proteins (Mm.386907 and Mm.87328) on the surface and the Mm.157049 protein in the intracellular region of mature sperm.

To further elucidate the distribution of the three novel proteins in mature sperm, sperm from the epididymis and vas deferens were fractionated into heads and tails, and their protein extracts were subjected to western blot analysis (Fig. 5B). ADAM2 and α-tubulin were used as control proteins localized in the heads and tails, respectively. The Mm.87328 protein was detected only in the head fraction. Unlike Mm.87328, the other two proteins (Mm386907 and Mm.157049) were found exclusively in the tail fraction (Fig. 5B). Taken together, our results disclosed that the Mm87328 protein is a sperm head molecule with cell surface localization, and the Mm.386907 and Mm.87328 proteins are sperm tail components residing at the cell surface (Mm.386907) and in an intracellular region (Mm.157049). We suggest to name the genes for these proteins *Shsp1 *(Sperm head surface protein 1 for Mm.87328), *Sfap1 *(Sperm flagellum associated protein 1 for Mm.386907) and *Sfap2 *(Sperm flagellum associated protein 2 for Mm.157049).

### Characterization of Shsp1/Mm.87328, Sfap1/Mm.386907 and Sfap2/Mm.157049

**Figure 6:**
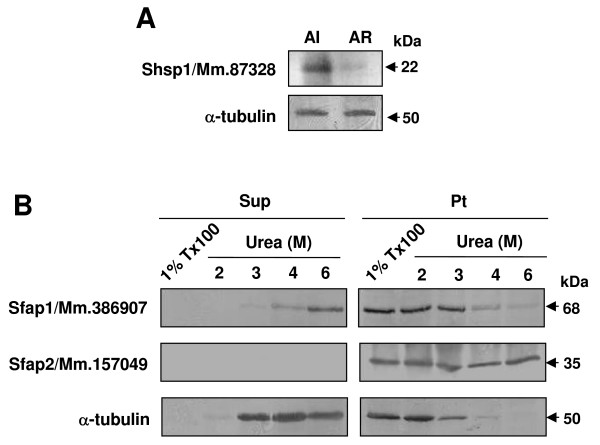
**Characterization of Shsp1/Mm.87328, Sfap1/Mm.386907 and Sfap2/Mm.157049.** (A) Acrosome reaction of sperm from the epididymis and vas deferens was induced by calcium ionophore A23187. Acrosome-intact and -reacted sperm were subjected to western blot anaylsis with the anti-Shsp1/Mm.87328 antibody. AI, acrosome-intact sperm; AR, acrosome-reacted sperm. (B) Sperm from the epididymis and vas deferens were treated with 1% Triton X-100 and urea with different concentrations (2, 3, 4 and 6 M). Soluble and insoluble fractions after centrifugation of the treated sperm were subjected to western blot anaylsis with the anti-Sfap1/Mm.386907 and anti-Sfap2/Mm.157049 antibodies. Sup, supernatant after centrifugation; Pt, pellet after centrifugation.

To further examine the characteristics and localization of Shsp1/Mm.87328, we compared the protein between acrosome-intact and -reacted sperm. During acrosome reaction, plasma membrane overlying the acrosome fuses with outer acrosomal membrane and consequently the two membranes are lost from the sperm head. We found that Shsp1/Mm.87328, present in acrosome-intact sperm, is absent from acrosome-reacted sperm (Fig. 6A). Thus, together with the finding on subcellular localization (Fig. 5A), this suggests that Shsp1/Mm.87328 is localized on the plasma membrane of the acrosomal region.

The unique cytoskeletal structures of sperm tail are outer dense fibers (ODFs) and fibrous sheath (FS). Proteins composing these structures exhibit similar solubility to various detergents. To explore the possibility that sperm tail-specific Sfap1/Mm.386907 and Sfap2/Mm.157049 are associated with these structures, we examined the extractability of the proteins using Triton X-100 and urea (Fig. 6B). Neither protein was solubilized with 1% Triton X-100. In sperm treated with varying concentrations (2, 3, 4 and 6 M) of urea, Sfap1/Mm.386907 was resistant to lower concentrations, and gradually solubilized with increasing concentrations. Sfap2/Mm.157049 was found to be completely resistant to extraction by 6 M urea (Fig. 6B). Thus, our findings imply that the two sperm tail proteins are the components of the cytoskeletal structures such as ODFs and FS of sperm flagellum or are firmly associated with them (see Discussion).

## Discussion

In our previous study, a number of novel genes exclusively expressed in testicular spermatogenic cells were discovered from the McCarrey Eddy round spermatid UniGene library (Lib. 6786), one of the largest mouse spermatogenic cell libraries deposited in the UniGene database at NCBI [1]. In the study, we utilized systematic, *in silico *and *in vitro *approaches, narrowing down all of the genes composing the library to 28 authentic genes with evident and abundant expression specific to testicular germ cells. Thus, it should be noted that these genes are most prominent among testicular novel genes and thereby deserve preferential investigation. Eight of the 28 genes were analyzed at the protein level in the present study.

In this study, we corroborated the authenticity of the eight genes at the protein level and further investigated the various properties of proteins encoded by the genes. Specific antibodies against the novel proteins were generated and they explicitly recognized distinctive bands, with the specific expected sizes of the proteins in the testicular spermatogenic cells. In the analysis of the germ cell-lacking testes of the *W/W*^*v *^mutant mice and Sertoli cells, the eight proteins were confirmed to be germ cell specific. The protein signals apparently were absent from the mutant mice. Further, we obtained new information about the developmental distribution of the novel proteins during spermatogenesis. Four of the proteins (Mm.307084, Mm.57415, Mm.67234, and Mm.380183) were detected only in the testicular germ cells, one protein (Mm.45611) in testicular germ cells and testicular sperm, and three proteins (Mm.87328, Mm.386907 and Mm.157049) in all the phases of sperm development and maturation, including testicular germ cells, testicular sperm and mature sperm.

Since many spermatogenic cell-specific genes have been shown to play critical roles in spermatogenesis and fertilization, it is possible that the present novel proteins are involved in these reproductive processes. The Mm.307084, Mm.57415, Mm.67234, Mm.380183 and Mm.45611 proteins could be involved in sperm development but not, at least directly, in sperm function and fertilization because they are absent from mature sperm. Our findings on the five proteins will expedite future investigation into these proteins, focusing on the developmental stages during which the proteins are present and active.

Our remarkable finding in the present study was the discovery of three novel proteins located at the head and tail of mature sperm. We designate these proteins as Shsp1/Mm.87328, Sfap1/Mm.386907 and Sfap2/Mm.157049, based on their specific distribution in sperm. Shsp1/Mm.87328 was found to be restricted to the head region of mature sperm, particularly the plasma membrane of the anterior part, covering the acrosomal region. The acrosome is a secretory organelle located at the apex of mature sperm. During fertilization, acrosome-intact sperm pass through cumulus cells and reach the egg extracellular coat, the zona pellucida (ZP). Upon recognition of the ZP and adhesion to it, sperm are induced to acrosome react. During acrosome reaction, the membrane surrounding the acrosome fuses with the plasma membrane of the sperm, exposing the contents at the site of sperm-egg ZP binding. It should be considered that proteins with localization patterns similar to that of Shsp1/Mm.87328 are candidates for participation in sperm-egg binding. Indeed, ADAM3, with Shsp1/Mm.87328-like distribution pattern on the sperm head, has been found to be critical for sperm adhesion to the egg ZP [6-9].

On the other hand, Sfap1/Mm.386907 and Sfap2/Mm.157049 were restricted to the tail region of mature sperm. The flagellum of mammalian sperm is divided into middle, principal and end pieces. The central core of the flagellum consists of a cytoskeletal structure called the axoneme. In the middle and principal pieces, the axoneme is surrounded by a unique structure, ODFs. The ODFs are enclosed by a helical array of mitochondria in the middle piece, while ODFs in the principal piece are surrounded by another sperm-specific structure, FS. ODFs and FS are considered to stiffen the sperm tail while allowing elastic bending. A common feature of proteins composing ODFs and FS is their resistance to non-ionic detergents. Furthermore, FS proteins are resistant to 6 M urea [10]. Our extraction analysis showed that Sfap1/Mm.386907 and Sfap2/Mm.157049 are found in the Triton X-100-insoluble fraction. Sfap1/Mm.386907 was solubilized with increasing concentrations of urea, with almost complete solubilization achieved at 6 M urea. Sfap2/Mm.157049 exhibited poor solubility, even in 6 M urea. This finding suggests that Sfap1/Mm.386907 is an ODF component or is weakly associated with FS, and Sfap2/Mm.157049 is an intrinsic component of FS. It is interesting to note that Sfap1/Mm.386907 was also found to be located at the sperm surface. This suggests that Sfap1/Mm.386907 is a transmembrane protein with a cytoplasmic region associated with the cytoskeletal structures in the sperm tail.

It should be noted that only a handful of ODF and FS proteins have been characterized at the protein and cellular levels [11-13]. Some of these proteins include ODF1 [14], ODF2 [15], SHIPPO 1 [16], Tektin 4 [17], protein kinase-A anchoring protein 3 and 4 [18,19], testis A-kinase anchoring protein [20], rhophilin [21], ropporin [22], spermatogenic cell-specific glyceraldehyde 3-phosphate dehydrogenate [23], and spermatogenic cell-specific hexokinase 1 [24]. These proteins are implicated in sperm structure, metabolism, motility and maturation [11,25,26]. Since abundant questions with respect to sperm tail proteins still remain, the identification of Sfap1/Mm.386907 and Sfap2/Mm.157049 will facilitate future studies on relationships of the sperm tail proteins with sperm function and fertilization.

## Conclusion

Male germ cell-specific genes are considered to play critical roles in spermatogenesis and fertilization. In this study, through the analyses of proteins encoded by eight novel genes expressed uniquely in spermatogenic cells, we provide new and inclusive information about authenticity, germ cell specificity, developmental expression patterns, and cellular localization of the eight novel proteins. In particular, the three proteins found to be localized in mature sperm are of greatest interest because of their cellular properties and potential functions. Our study will help to determine molecular mechanisms of male reproduction involving these novel proteins.

## Competing interests

The authors declare that they have no competing interests.

## Authors' contributions

NB, J–MW and CH contributed equally to this work. NB, J–MW and CH generated antigens and antibodies. NB, J–MW, CH, EC and IP performed western blot analysis. DHK, EME and CC conceived and directed the project. NB and CC designed the study and drafted the manuscript. All authors read and approved the final manuscript.
